# Current surgical instrument labeling techniques may increase the risk of unintentionally retained foreign objects: a hypothesis

**DOI:** 10.1186/1754-9493-7-31

**Published:** 2013-09-30

**Authors:** Kyros Ipaktchi, Adam Kolnik, Michael Messina, Rodrigo Banegas, Meryl Livermore, Connie Price

**Affiliations:** 1 Department of Orthopaedic Surgery, Denver Health Medical Center, 777 Bannock Street, Denver, CO 80204, USA; 2 Department of Medicine, Infectious Diseases, Denver Health Medical Center, 777 Bannock Street, Denver, CO 80204, USA; 3 University of Colorado School of Medicine, Anschutz Campus, Aurora, CO 80045, USA

**Keywords:** Surgical instrument marking, Instrument identification, "Never event"

## Abstract

**Background:**

Marking of surgical instruments is essential to ensure their proper identification after sterile processing. The National Quality Forum defines unintentionally retained foreign objects in a surgical patient as a serious reportable event also called "never event."

**Presentation of the hypothesis:**

We hypothesize that established practices of surgical instrument identification using unkempt tape labels and plastic tags may expose patients to "never events" from retained disintegrating labels.

**Testing of the hypothesis:**

We demonstrate the near miss of a "never event" during a surgical case in which the breakage of an instrument label remained initially unwitnessed. A fragment of the plastic label was accidentally found in the wound upon closing. Further clinical testing of the occurrence of this "never event" appears not feasible. As the name implies a patient should never be exposed to the risk of fragmenting labels.

**Implication of the hypothesis:**

Current practice does not mandate verifying intact instrument markers as part of the instrument count. The clinical confirmation of our hypothesis mandates a change in perioperative practice: Mechanical labels need to undergo routine inspection and maintenance. The perioperative count must not only verify the quantity of surgical instruments but also the intactness of labels to ensure that no part of an instrument is left behind. Proactive maintenance of taped and dipped labels should be performed routinely. The implementation of newer labeling technologies - such as laser engraved codes - appears to eliminate risks seen in traditional mechanical labels.

This article reviews current instrument marking technologies, highlights shortcomings and recommends safe instrument handling and marking practices implementing newer available technologies.

## Background

The specialization of surgical practice exponentially increased the amount of instruments a modern surgical center utilizes. It has been calculated that a surgical center maintains at any time several tens of thousands of instruments and that each center on average processes millions of instruments per year [1]. Instruments are transported between surgical suites and sterile processing units and may be shipped to satellite facilities of major institutions. The proper labeling of instruments is essential to identify each tool, to restock instrument sets and to maintain oversight of instrument stock.

**Figure 1:**
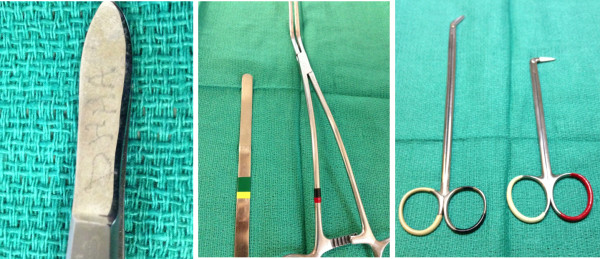
Traditional Instrument Marking Techniques: Left panel – Instrument labeling using etching techniques which disrupts instrument surface; Center panel - Instrument body wrapped with surgical instrument label tape; Right panel – Instrument ends dipped in plastic coating in various stages of degradation.

Traditionally, instruments have been identified by various engraving and etching techniques (Figure 1 Left panel). Given the subtleties of finer surgical instruments and the demand for smooth surfaces, this crude form labeling has been largely abandoned in the last decades in favor of tape labeling or plastic resin dipping of instruments (Figure 1 Center panel/Right panel). These techniques leave the instrument surface intact and add the benefit of color coding. There are several downsides to this practice which have been in part discussed in literature [2]. Any mechanical labeling of an instrument needs to be supervised by a professional surgical technologist to ensure proper label application. The label must withstand the mechanical load of intraoperative utilization as well as the physico-chemical challenges of cleaning and sterilization cycles [3]. In addition to time and resources spent initially labeling the tool, there needs to be continuous maintenance to insure intactness of the label.

### Presentation of the hypothesis

Retained surgical instruments are considered by the National Quality Forum (NQF) as serious reportable events or "never events" [4,5]. We argue that instrument labels are part of a surgical instrument. We hypothesize that retained fragments of a surgical instrument label expose patients to a "never event".

### Testing of the hypothesis

**Figure 2:**
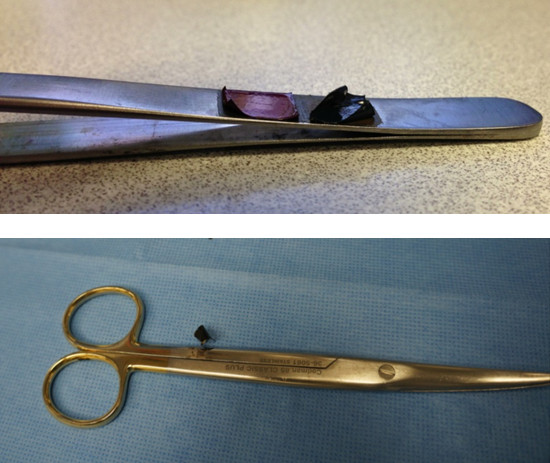
Defective Labels: Top panel - Delaminating tape label peeling off a surgical forceps; Bottom panel - Fragmented plastic coating which broke off the end of a scissor during a surgical case.

We demonstrate the occurrence of a near missed "never event" in the form of an intraoperative break of a surgical label, additionally we found delaminated tape labeling material during a review of our instrument sets on a microsurgical instruments (Figure 2 Top panel). Label fragmentation, demonstrated on a surgical scissor during a surgical case may end up as an unaccounted residual foreign object in the patient (Figure 2 Bottom panel). In the case of the label failure in Figure 2 Bottom panel, the disintegration of the label itself escaped the attention of the team. At the end of the case the surgical team accidentally found and retrieved a foreign body in the wound before closing. Upon inspection the foreign object turned out to be an instrument label. The ensuing immediate assessment of all instruments demonstrated a defective scissor label as origin of the foreign object. This "never event" was fortuitously averted as the surgical team accidentally detected and retrieved the label fragment. As implied by the name: "never events" should never occur. The demonstration such an event supports our hypothesis and does not warrant further testing.

### Implication of the hypothesis

Retained instrument labeling is an avoidable "never event". Mechanical labels need routine maintenance to verify their intactness. Loose or fragile labels can expose the patient and the surgical team to the risk of intraoperative breakage. Retained labels in a surgical wound may remain unaccounted and undetectable. Intraoperative instrument verification and counting must include instrument labels. We recommend the utilization of laser engraved encoding of instruments which offers the benefit of being non-detachable and allowing state of the art instrument tracking. Literature describes several cases of broken instrument labels: A case from 1983 reports a 1 × 0.5 cm piece of marking tape identified and subsequently removed in the repair of an oro-antral fistula [2]. This piece originated from a curette. The authors also discuss the occurrence of abscesses following mandibular skin graft vestibuloplasty in 4 out of 6 patients undergoing the procedure. Cultures revealed staphylococcus epidermidis, which was traced back to two loose pieces of marking tape on awl handles. The second case reports on an elective tracheostomy complicated by subsequent clot obstruction of the left main bronchus [6]. Authors concluded that the clot formed in reaction to a piece of colored marking tape eroding through the mucosa of the left main bronchus; the tape had originated from a surgical instrument. These case reports, led to a recommendation by the Food and Drug Administration (FDA) in 1996 to perform a survey of operating room nurse managers to assess "tape durability, extent of use, and whether there are any practices or procedures for marking surgical instruments and/or any human factors that could be altered to better protect the public health" [7]. It is unclear to the authors whether this survey was ever conducted. There is no further mention of it in the FDA database.

In July 2012, the FDA proposed the introduction of regulation requiring all medical devices to be labeled with a unique device identifier (UDI) to allow tracking with automatic identification and data capture (AIDC) technology. Under the proposed rule, information about devices labeled with a UDI will be available to the health care community and public through the Global Unique Device Identification Database (GUDID) [5]. A staged implementation of surgical instrument marking with UDIs is proposed: One year after the publication of the final regulation, all class III medical devices (e.g. pacemakers, heart valves) will be required to be UDI - labeled. Class II medical devices, including surgical instruments, will under this proposition be UDI - labeled three years later. It thus appears likely that modern labeling techniques such as laser engraving will replace mechanical labeling for both safety and regulatory reasons. Outside the United States, the need for continuous instrument tracking is also recognized and mandated by according directives regulating medical device usage within the member countries of the European Community [8].

With regards to labels being potential sources for infection, the evidence is equivocal. An experimental study demonstrated the effectiveness of flash sterilization in removing an experimental inoculation with bacillus stearothermophilus between strips of colored marking tape and the surface of surgical instruments [9].

We argue that labels are part of a surgical instrument and that retained label fragments represent a surgical "never event" as defined by the National Quality Forum (NQF) [4,5,10,11] (see also Table 1). We argue that label intactness needs to be accounted for as part of the final instrument count [12]. A special problem of retained labels appears to be the fact that they are radiolucent: Once retained they will escape standard radiographic detection.

**Table 1:** Surgical "never events" according to the National Quality Forum (NQF) consensus report (2006)

	**Serious reportable surgical events "never events"**
1	Surgery performed on wrong surgical site
2	Surgery performed on wrong patient
3	Wrong surgical procedure
4	Unintentionally retained foreign object in a surgical patient
5	Intraoperative or immediate postoperative death in a ASA class 1 patient

**Figure 3:**
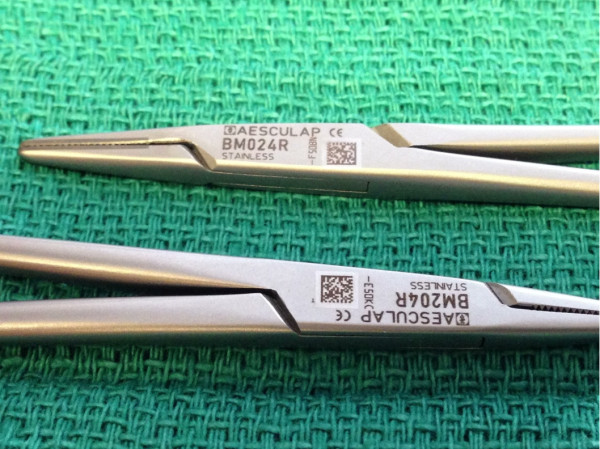
Modern Quick response (QR) Code Labeling: Low profile modern labeling techniques which do not disrupt instrument surface offering machine readable, electronic tracking options.

Modern labeling techniques such as laser engraved two dimensional Quick Response (QR) codes or one dimensional bar coding offer alternatives to mechanical labeling (Figure 3). Laser engraved, machine readable labels offer a durable tagging technique with no macroscopic new interface. In addition, these labels allow real time tracking of instrumentation processing cycles and adherence to maintenance protocols.

## Abbreviations

NQF: National Quality Forum; FDA: Food and Drug Administration; QR code™: Quick Response Code, Denso ADC 1994.

## Competing interests

The authors declare that they have no competing interests. There is no conflict of interest in regards to the displayed vendor name on Figure 3.

## Authors’ contributions

KI: Conception, first draft of manuscript, provision of images. AK, CP, ML, RB: Critical review of manuscript. All authors read and approved the final manuscript.
